# Investigation of gray matter volume in individuals with heart failure and preserved ejection fraction

**DOI:** 10.3389/fnagi.2025.1486381

**Published:** 2025-06-13

**Authors:** Tianyi Yu, Qiuyun Bai, Yiting Guo, Yuchun Yuan

**Affiliations:** ^1^Department of Radiology, Shandong Provincial Hospital Affiliated to Shandong First Medical University, Jinan, China; ^2^Department of Radiology, The Second Affiliated Hospital of Shandong University of Traditional Chinese Medicine, Jinan, China

**Keywords:** heart failure with preserved ejection fraction, gray matter volume, MRI, VBM, cognitive function

## Abstract

**Object:**

This study employs voxel-based morphometry techniques to identify potential areas of brain injury in patients with heart failure with preserved ejection fraction (HFpEF). It further assesses the correlation between clinical indicators, cardiac function parameters, and gray matter volume (GMV). This provides an imaging-based anatomical biomarker for in-depth research into the brain structure in patients with HFpEF.

**Materials and methods:**

This study recruited 51 patients with HFpEF (26 males and 25 females) and 40 healthy controls (27 males and 13 females). Data on NT-proBNP levels, echocardiographic parameters, and cognitive function scores were collected for both groups. High-resolution 3D T1-weighted imaging (3D-T1WI) structural MRI data were collected from all participants. The changes in GMV between the two groups were assessed using voxel-based morphometry (VBM).

**Results:**

The study involved 40 patients with HFpEF and 28 healthy controls (HC). No significant differences were observed between the groups regarding age, gender, education, or BMI. The HFpEF group exhibited larger measurements for Left Ventricular Posterior Wall (LVPW), Interventricular Septal Thickness (IVST), Left Atrial Diameter (LAD), Right Atrial Diameter (RAD), and Right Ventricular Diameter (RVD). However, they maintained preserved systolic function and achieved lower scores on the MoCA, indicating deficits in visuospatial/executive functions, naming, attention, language, and memory. Compared to HC, HFpEF patients had reduced GMV in specific brain regions. NT-proBNP levels were negatively correlated with GM reduction in various cerebellar, frontal, temporal, and postcentral regions. Cognitive performance was inversely related to GM shrinkage, with different brain regions correlating with specific cognitive deficits.

**Conclusion:**

Abnormalities in GMV in several brain areas have been identified in patients with HFpEF. Furthermore, these abnormal GMV are associated with NT-proBNP levels, echocardiographic indices, and neurocognitive scoring. These observations could provide fresh perspectives on the pathogenic mechanisms of HFpEF.

## 1 Introduction

Heart failure (HF) comprises a range of syndromes marked by various structural or functional cardiac disorders. These impairments lead to difficulties in ventricular filling and/or ejection, causing cardiac output to be inadequate for meeting the metabolic needs of body tissues. Consequently, this results in insufficient blood flow to the lungs and other organs and tissues (Hanon et al., 2014). The 2016 European Society of Cardiology guidelines for heart failure stratify the condition into three categories based on Left Ventricular Ejection Fraction (LVEF): HFrEF is defined as LVEF < 40%, while Heart Failure with Mid-Range Ejection Fraction (HFmrEF) applies to LVEF between 40 and 49%. Heart Failure with Preserved Ejection Fraction (HFpEF) is characterized by an LVEF of 50% or greater (Savarese et al., 2022). HFpEF, in particular, is a complex and phenotypically diverse syndrome featured by ventricular diastolic dysfunction coupled with high end-diastolic pressure, while maintaining a normal or near-normal LVEF, thus is also known as Diastolic Heart Failure (DHF) (Eltelbany et al., 2022). Previous studies have reported cognitive impairment (CI) in patients with HF, particularly affecting processing speed, verbal memory, and executive function (Cui et al., 2020). Brain injury may be the main contributor to these clinical symptoms, and changes in the brain’s gray matter (GM) structure have been reported in HF patients (Steinberg et al., 2012). The centers for cognitive and executive functions are concentrated in the cortical structures of the brain (Zhou et al., 2021), which are highly sensitive to hypoxia, with irreversible damage occurring if oxygen deprivation exceeds 4–5 min. Therefore, recognizing the changes in the anatomical structure of the brain’s GM is crucial for understanding the cognitive dysfunction associated with HFpEF.

Since 2000, voxel-based morphometry (VBM) has been increasingly applied in the study of neuropsychiatric disorders (Whitwell, 2009). VBM is an automated, voxel-wise method for analyzing neuroanatomy, utilizing statistical techniques to process high-resolution three-dimensional magnetic resonance imaging (MRI) data. This approach enables precise detection and quantification of local gray and white matter density and volume. A key advantage of VBM is its capacity to identify subtle structural changes in the brain without requiring predefined regions of interest, thus minimizing operator bias. This high sensitivity makes VBM particularly effective for detecting diseases associated with neuropsychological dysfunction before any macroscopic structural changes occur (Good et al., 2001; Ridgway et al., 2008).

This study employs voxel-based morphometry to identify potential brain injury regions in HFpEF and to assess the associations between clinical indicators, cardiac functional parameters, and gray matter volume (GMV), thereby providing an anatomical biomarker via imaging for further investigation into the cerebral function of HFpEF.

## 2 Method

The experimental subjects included a total of 51 subjects in the HFpEF group (26 males and 25 females), and 40 members in the HC group (27 males and 13 females). Patients enrolled in this study met the diagnostic criteria for HFpEF, exhibiting typical signs and symptoms of heart failure, with BNP ≥ 35 pg./mL or NT-proBNP > 125 ng/L. Echocardiographic examination demonstrated structural cardiac abnormalities and/or impaired diastolic or systolic function, with LVEF ≥ 50%. Eligible participants were right-handed individuals aged between 45 and 80 years who provided written informed consent and were willing to cooperate with the study procedures.

Exclusion criteria included acute exacerbation of heart failure within the past 2 months, unstable cardiovascular or cerebrovascular diseases, dementia, uncontrolled hypertension, psychiatric disorders, a history of traumatic brain injury or brain tumor, obstructive sleep apnea, severe metabolic diseases (such as hepatic or renal failure, or decompensated diabetes mellitus), alcohol or substance dependence, illiteracy, and epilepsy. These exclusion criteria were also applied to the healthy control (HC) group.

### 2.1 Clinical data

All patients fasted overnight for at least 8 h before venous blood samples were collected the following day for analysis. The tests included serum hypersensitive C-reactive protein (hs-CRP), fasting plasma glucose (FPG), triglycerides (TG), total cholesterol (TC), and N-terminal pro-brain natriuretic peptide (NT-proBNP). All aforementioned laboratory tests were carried out by the Department of Laboratory Medicine at the Affiliated Provincial Hospital of Shandong First Medical University.

All patients underwent echocardiographic examinations at the Echocardiography Laboratory of the Affiliated Provincial Hospital of Shandong First Medical University, where the following data were collected and recorded: LVEF, left ventricular end-diastolic diameter (LVDD), left atrial diameter (LAD), interventricular septal thickness (IVST), left ventricular posterior wall thickness (LVPWT), right atrial transverse diameter (RAD), and right ventricular anteroposterior diameter (RVD).

### 2.2 Neuropsychological examinations

The participants in the study were evaluated for their cognitive status using the Montreal Cognitive Assessment (MoCA) scales (Nasreddine et al., 2005). The MoCA scale evaluates a range of cognitive functions, including visuospatial and executive abilities, naming skills, attention, language proficiency, abstraction, memory recall, and orientation. These assessments were conducted following standardized procedures in a quiet environment. The maximum score for both scales is 30 points. Scores below 26 on the MoCA indicate poor cognitive function.

### 2.3 Magnetic resonance imaging protocol

Whole-brain images were obtained at the Shandong Provincial Hospital Affiliated to Shandong First Medical University using a Siemens 3.0 T Prisma MR system and a 64-channel head coil for brain scanning. Participants were carefully positioned inside the machine, and foam padding was used to minimize any movement during the scanning process.

T1-weighted whole-brain magnetization prepared rapid acquisition gradient echo imaging were collected to capture anatomical details using the following parameters: TR = 2,530 ms, TE = 2.98 ms, TI = 1,100 ms, FOV = 256 × 256 mm^2^, in-plane resolution = 256 × 256 mm^2^, flip angle = 7°, and 192 axial slices.

All MRI images were reviewed by two senior neuroimaging diagnosticians.

### 2.4 Data preprocessing

The preprocessing of all 3D-T1WI structural image data was performed using the CAT12 software within the SPM12 (based on the Matlab 7.10 platform). The main process is as follows: First, MRI data were converted from DICOM format to NIFTI format using the MRIcron software. The 3D T1-weighted structural images of the entire brain were then bias-corrected and segmented in GM, white matter, and cerebrospinal fluid. Subsequently, the GM images were affine registered to the standard brain template of the Montreal Neurological Institute (MNI) and a study-specific template was created for this tissue utilizing the Diffeomorphic Anatomical Registration Through Exponentiated Lie (DARTEL) algorithm. The original GM images were then spatially registered to the newly generated template and further normalized to the MNI space (with isotropic voxels of 1.5 mm). The resulting GM images were modulated using the Jacobian determinants to account for volume changes. Finally, the modulated GM images were smoothed with an isotropic Gaussian kernel that had a full width at half maximum of 8 mm.

### 2.5 Statistical analysis

Data analysis was carried out using SPSS 22.0 statistical software. Quantitative data were expressed as mean ± standard deviation (x̄ ± s). The two-sample t-test was used to evaluate differences between the two groups in terms of age, education level, BMI, MoCA scores, and clinical indicators. Gender differences were assessed using the chi-square test. GMV between the two groups was compared using two-sample t-tests with SPM8 statistical software, including each subject’s gender, age, education level, and BMI as covariates. Clusters were set to a minimum size of 200 voxels, and statistical results were corrected using the Gaussian Random Field Theory (GRF), with a significance level of *p* < 0.05 indicating regions of GM with significant differences. The relationship between VBM values and MoCA scores, cardiac function, and laboratory tests were examined using Pearson correlation analysis, with *p* < 0.05 considered statistically significant.

## 3 Results

In this study, a total of 51 patients with HFpEF participated. However, 11 individuals were excluded for the following reasons: 3 voluntarily withdrew from the study, 2 were excluded due to severe image artifacts caused by head movement, 3 had multiple lacunar strokes, 2 suffered from claustrophobia, and 1 had an arachnoid cyst. Consequently, 40 patients were included in the data analysis. As for the HC, 40 individuals initially participated, but exclusions were made as follows: 3 voluntarily withdrew, 2 were found to have mild cognitive impairment (MCI), 4 were excluded due to head movement artifacts, and 3 had multiple lacunar strokes, leaving 28 participants included in the study.

### 3.1 Comparison of clinical data between HFpEF group and HC group

There were no statistically significant differences in age, gender, education level, and BMI between the HFpEF group and the HC group (*p* > 0.05). The HFpEF group exhibited significantly higher values of LVPW, IVST, LAD, and RAD compared to the HC group, with no notable impairment in systolic function observed. The MoCA scores of the HFpEF group were significantly lower than those of the HC group, revealing CI primarily in the areas of visuospatial and executive functions, naming, attention, language, and memory (*p* < 0.05). In contrast, no statistical significance was found in abstract thinking and orientation (*p* > 0.05) (Table 1).

**Table 1:** Comparison of demographic data between HFpEF group and HC group.

Variable	HF	HC	*P*
	40	28	
Age (years), mean (SD)	60.20 ± 7.91	60.42 ± 7.99	0.90
Male sex, No. (%)	24 (60%)	17 (61%)	0.95
Education, years	10.75 ± 4.36	9.85 ± 3.21	0.36
BMI (Kg/m^2^)	25.93 ± 2.85	25.05 ± 1.22	0.13
Smokers (*n*%)	14 (35%)	11 (39%)	0.72
Drinkers (*n*%)	15 (38%)	12 (43%)	0.66
Hypertension (*n*%)	23 (58%)	15 (54%)	0.75
Dyslipidemia (*n*%)	11 (27.5%)	9 (32%)	0.68
Diabetes mellitus (*n*%)	6 (15%)	7 (25%)	0.30
MOCA Score	22.95 ± 3.54	27.32 ± 2.69	0
NT-proBNP (pg/mL)	283.33 ± 241.04	78.69 ± 130.84	0.0001
CRP	2.03 ± 2.31	3.42 ± 8.13	0.38
Glucose	5.09 ± 1.32	5.22 ± 0.83	0.61
Total cholesterol	4.34 ± 0.95	4.59 ± 0.92	0.28
Triglyceride	1.36 ± 0.44	1.74 ± 0.92	0.05
LVEF	59.9 ± 2.91	61.75 ± 1.81	0.0041
LVDD	4.90 ± 0.45	4.67 ± 0.43	0.03
LVPW	0.98 ± 0.10	0.86 ± 0.14	0.0002
IVST	0.99 ± 0.11	0.88 ± 0.15	0.0016
LAD	4.08 ± 0.54	3.24 ± 0.42	0
RAD	4.21 ± 0.57	3.49 ± 0.56	0
RVD	2.08 ± 0.23	2.27 ± 0.45	0.04

### 3.2 Comparison of gray matter volume between HFpEF group and HC group

**Figure 1:**
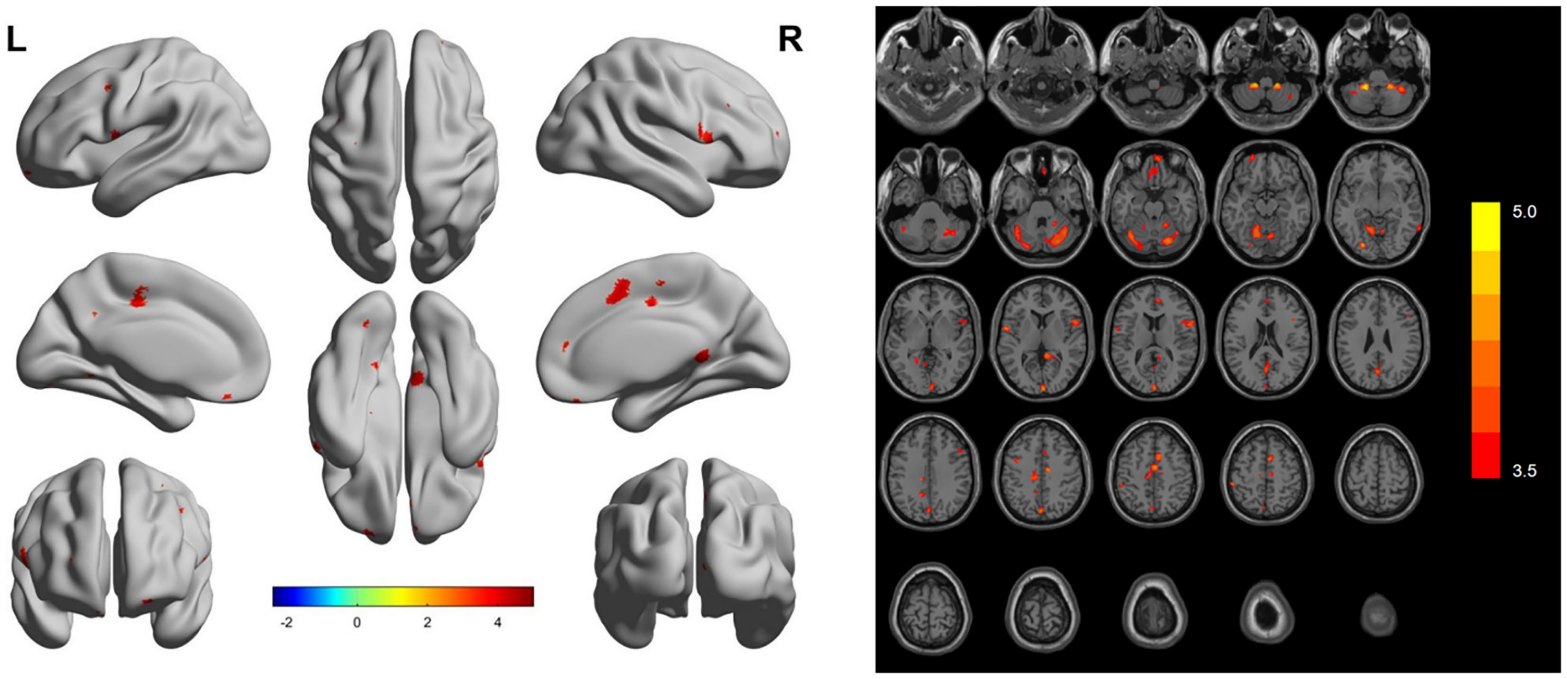
Differences in gray matter volume between the HFpEF group and the HC group. The two images represent the differences of gray matter volume between HFpEF group and HC group (The red area represents the area where the gray matter volume increases).

In the HFpEF group, regions of decreased GMV were observed in the bilateral cerebellar hemispheres, right posterior cingulate gyrus, right inferior frontal gyrus, right supplementary motor area, bilateral middle frontal gyri, right middle temporal gyrus, right superior frontal gyrus, left calcarine fissure and adjacent cortex, left inferior frontal gyrus, left superior frontal gyrus, and left postcentral gyrus (Figure 1, Table 2).

**Table 2:** Comparative analysis of gray matter volume between HFpEF group and HC group.

Regions	Hemi	AAL	MNI coordinates	Volume (mm^3^)	*T*
Cerebellar hemisphere	R	100	18, −39, −49.5	10202.6	4.6576
Cerebellar hemisphere	L	91	−24, −81 −4.5	5666.62	4.6243
Posterior cingulate gyrus	R	36	7.5, −42, 7.5	4363.88	4.1102
Inferior frontal gyrus	R	12	55.5, 12, 3	2561.62	4.0094
Supplementary motor area	R	20	6, 7.5, 52.5	1778.62	4.3156
Calcarine fissure and surrounding cortex	L	43	1.5, −96, 9	1768.5	4.4721
Superior frontal gyrus, medial orbital	R	26	11, 61, −21	1549.12	3.6469
Inferior frontal gyrus, opercular part	L	11	−58.5, 1.5, 7.5	955.125	4.2078
Middle frontal gyrus	L	7	−22.5, 58.5, −13.5	590.625	3.5885
Superior frontal gyrus, medial	L	23	3, 46.5, 16.5	573.75	3.7497
Postcentral gyrus	L	57	−54, −33, 54	486	4.3503
Middle frontal gyrus	R	8	48, 22.5, 31.5	438.75	3.8014
Middle temporal gyrus	R	86	67.5, −55.5, −6	384.75	3.7726

### 3.3 Correlation analysis

In this study, NT-proBNP levels were negatively correlated with decreased GMV in several brain regions, including the right and left cerebellar hemispheres, right posterior cingulate gyrus, right inferior frontal gyrus, right supplementary motor area, right and left middle frontal gyri, right superior frontal gyrus, left superior frontal gyrus, left postcentral gyrus, and right middle temporal gyrus. These findings suggest that reductions in GMV in these regions are associated with cognitive dysfunction in HFpEF patients.

The shrinkage of GM in the right cerebellar hemisphere was negatively correlated with performance in naming, attention, and language tasks. A similar reduction in the left cerebellar hemisphere was negatively associated with attention, language, and memory scores. Decreased GM in the right posterior cingulate gyrus was negatively correlated with naming and language scores, while the right inferior frontal gyrus showed a negative correlation with naming, language, and memory scores. The shrinkage of the right supplementary motor area was negatively related to language performance.

Additionally, reductions in GMV in the right superior frontal gyrus were negatively correlated with memory scores. The volume reduction in the left inferior frontal gyrus was associated with attention and language scores, and the reduction in the left middle frontal gyrus was negatively correlated with naming and language scores. The left superior frontal gyrus volume reduction was negatively associated with attention and language scores, while the left postcentral gyrus showed a negative correlation with attention and language scores. Finally, the reduction in the right middle temporal gyrus was negatively correlated with naming scores.

**Figure 2:**
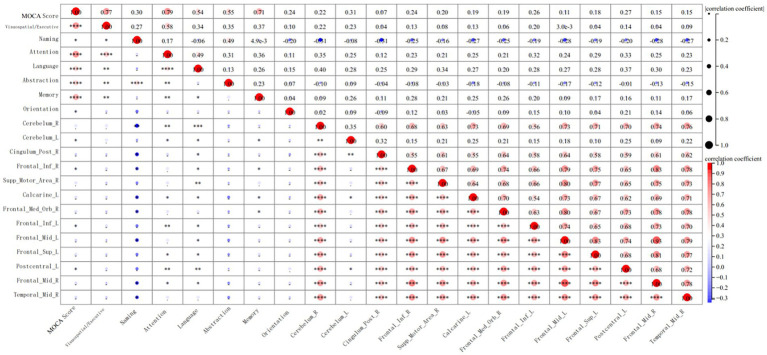
Correlation analysis between gray matter volume and cognitive function in HFpEF group.

**Figure 3:**
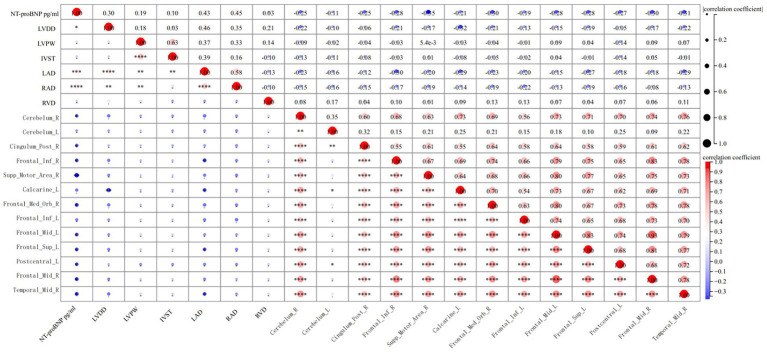
Correlation analysis between gray matter volume and clinical indexes in HFpEF group.

Figures 2, 3 illustrate these significant negative correlations between GMV reductions and cognitive function scores across the different brain regions.

## 4 Discussion

This study utilized VBM technology to assess alterations in GM structure within HFpEF patients. Compared to the HC group, HFpEF individuals displayed reduced GMV in several areas, including the bilateral cerebellar hemispheres, right posterior cingulate gyrus, right inferior frontal gyrus, right supplementary motor area, bilateral middle frontal gyri, right middle temporal gyrus, right superior frontal gyrus, left calcarine fissure and adjacent cortex, left inferior frontal gyrus, left superior frontal gyrus, and left postcentral gyrus. The HFpEF group scored significantly lower on the MoCA, particularly in the domains of visuospatial and executive functions, naming, attention, language, and memory. Reductions in GMV in the right cerebellar hemisphere, right inferior frontal gyrus, right supplementary motor area, cortex surrounding the left calcarine fissure, right medial orbitofrontal gyrus, left superior frontal gyrus, and right middle temporal gyrus were associated with abnormalities in cardiac and cognitive functions.

The findings of this study are not completely consistent with previous reports on changes in GMV in patients with heart failure. However, the study confirms the presence of neuronal damage in HFpEF patients and further reveals the brain regions affected by the impaired GM structures. These brain areas play an important role in key cognitive domains such as executive functions, memory, and naming. The decreased GMV in the right and left cerebellar hemispheres may affect motor control and balance regulation. The cerebellum is a key structure for coordinating movement and maintaining postural balance, and its dysfunction could lead to motor incoordination and balance disorders, which in turn affect the executive aspects of cognitive function (Houghton et al., 2021). The frontal lobe is a crucial brain area for central functions like movement, memory, language, impulse control, and social behavior (Arain et al., 2013). Damage to or functional abnormalities in the right supplementary motor area may affect patients’ motor planning and execution abilities, leading to issues such as motor incoordination and difficulty with movement pre-setting (Ding et al., 2023). These motor-related problems may be closely related to common symptoms in heart failure patients, such as fatigue and decreased exercise endurance. Damage to or functional abnormalities in the cortex surrounding the left calcarine fissure may affect patients’ language abilities and motor control (Chu et al., 2023). This could lead to issues such as impaired speech fluency, difficulty with speech comprehension, and inflexibility in hand movements, affecting patients’ daily life and social interactions. The parietal and occipital cortices are also involved in cognitive and behavioral functions; the parietal cortex has the function of understanding, encoding, consolidating, and retrieving written language and manipulating working memory (Koenigs et al., 2009). The occipital lobe is involved in memory, visuo-constructional skills, calculation, and task execution (Brownsett and Wise, 2010). Meanwhile, hippocampal structures within the temporal lobe are involved in memory function (Csernansky et al., 2005). Therefore, cortical damage in these areas may result in functional changes, manifesting as abnormalities in cognitive behavior.

The decrease in brain GMV in HFpEF patients is generally caused by loss of neuronal cells or neuronal damage (Ahad et al., 2020). Reduced GMV in the brain indicates atrophy of brain tissue and damage at the vascular or cellular level, including neurodegenerative changes (Wang et al., 2017). Initially, HF patients’ brain structures may show no global atrophy, with only the integrity of white matter fiber structures being impaired (Vogels et al., 2007). Current research on localized GMV reduction is not sufficiently in-depth, and many study results are inconsistent (Frey et al., 2021). Some scholars have found (Ogoh et al., 2022) structural abnormalities in areas such as the frontal lobe and cerebellum in heart failure patients, suggesting that atrophy in these brain regions may play an important role in the CI of chronic heart failure patients. International studies using conventional MRI techniques, such as those by Kumar et al. (2009), have quantitatively measured the brain regions’ volumes, including the frontal lobe, hippocampus, and mammillary bodies, in heart failure patients, revealing significant brain atrophy in the mentioned areas. Although the results are not entirely consistent, they at least all suggest that chronic heart failure can lead to atrophy of the brain’s GM structure in patients. In experimental work exploring the pathogenesis of cognitive dysfunction, it is widely considered that differences in brain areas such as the frontal lobe, temporal lobe, caudate nucleus, and cerebellum play an important role. These differences may be attributed to the heterogeneity of the study populations, MRI acquisition techniques, voxel-based morphological analysis methods, as well as differences in statistical analysis and processing procedures.

Increasing evidence suggests that brain atrophy is not solely attributable to neurodegenerative mechanisms but is also closely associated with cerebrovascular factors (Ye et al., 2022). Chronic cerebral hypoperfusion, impaired cerebral autoregulation, and endothelial dysfunction are commonly observed in HF patients, potentially leading to both gray and white matter damage, thus providing a vascular basis for neuronal injury (Ni et al., 2023). Notably, periventricular and deep white matter hyperintensities (WMHs) are imaging manifestations of small vessel disease and are often assessed using the Fazekas scale. These lesions have been consistently linked to cognitive decline in multiple studies (Prins and Scheltens, 2015; Zhang et al., 2023). WMHs represent chronic ischemic damage, which may disrupt cortical–subcortical circuits, consequently affecting executive functions and memory—domains frequently impaired in HFpEF-related cognitive dysfunction. Although this study focuses on GMV changes, future research incorporating WMHs assessment could offer a more comprehensive understanding of the brain structural changes associated with HFpEF.

The reduction in brain GMV may stem from hypoxia-induced neuroinflammation and neuroglial cell damage, indicating an immune response in the affected GM areas (Brooks and Mias, 2019). Imaging technologies are key tools in identifying brain injury related to HFpEF, including advanced functional MRI techniques such as diffusion tensor imaging and magnetic resonance spectroscopy, which provide important information about local anatomical structure and neurochemical environmental changes for clinical use (Spilling et al., 2017). Pathological examination is considered the gold standard for diagnosing abnormalities in brain GMV, but due to the invasiveness of brain tissue biopsy and the potential damage to local GM function, it is not suitable for use in clinical trials (Lancaster et al., 2013). Moreover, the reduction in brain GMV also reflects structural changes in the local GM due to the long-term impact of disease. Brain function abnormalities in patients with HFpEF may lead to MCI, a condition that may remain undetected for many years. Therefore, assessing the duration of cognitive dysfunction or the affected time span of neuropsychological performance in HFpEF patients is crucial for understanding their brain structural changes. For example, although numerous studies have indicated hippocampal atrophy in patients with heart failure, this study did not find significant abnormal changes in hippocampal volume. Whether these subtle changes in GM nuclei affect cortical brain function remains unclear, hence, it is necessary to combine structural and functional analysis to further investigate changes in these brain regions.

The reduction in GMV in HFpEF patients may also result from common systemic factors that contribute to the aging of both the heart and brain. These include hypertension, diabetes, atrial fibrillation, and endothelial dysfunction. These shared pathologies may impair the integrity of both myocardial and cerebral vasculature, leading to compromised perfusion and tissue damage (Ye et al., 2022). Preventive strategies targeting vascular health, such as strict blood pressure control, physical activity, blood glucose management, and anti-inflammatory interventions, may help protect the structure and function of both the heart and brain (Liori et al., 2022; Mene-Afejuku et al., 2019). Therefore, cardiovascular-neurological integrated treatment could be crucial in delaying or preventing cognitive decline in HFpEF patients.

This study identified a negative correlation between NT-proBNP levels and volume reductions in various brain regions, such as the right cerebellar hemisphere, right posterior cingulate gyrus, right inferior frontal gyrus, right supplementary motor area, right superior frontal gyrus, left middle frontal gyrus, left superior frontal gyrus, left postcentral gyrus, right middle frontal gyrus, and right middle temporal gyrus. This negative correlation may reflect the impact of heart failure on these brain areas. Notably, these regions play an important role in functions such as movement and cognition; thus, NT-proBNP levels may serve as a biomarker for brain structural changes in patients with HFpEF. These findings further emphasize that heart failure not only affects cardiac function but may also have a negative impact on brain structure and function. Future research could further explore the mechanistic links between NT-proBNP and brain structural changes, as well as how this relationship affects the clinical presentations and prognosis of patients with heart failure.

### 4.1 Limitation

Our study does have certain limitations that should be acknowledged. The primary limitation lies in the small sample size, which could limit the generalizability of our findings and diminish the statistical power of our analysis. Additionally, the recruitment of study participants from a singular medical institution may introduce recruitment bias and may not accurately reflect the broader HF population. Therefore, further studies with larger and more diverse samples are needed to validate our results. Additionally, in terms of neuropsychological examination, we only utilized the MoCA scales to assess the cognitive function of the subjects. While these scales provide valuable insights, they may not comprehensively evaluate all aspects of cognitive decline. In future studies, it will be important to include a broader range of cognitive assessment tools to obtain a more precise understanding of the degree of decline in various cognitive functions. Furthermore, we did not compare brain MRI differences between HFpEF and HFrEF patients. Including such a comparison in future studies would help highlight the specific structural changes in HFpEF and enhance the novelty of the findings.

## 5 Conclusion

In conclusion, compared to the healthy control group, the HFpEF patient group showed cognitive decline and abnormal changes in gray matter volume in specific brain regions. These changes were closely linked to laboratory results, cardiac function, and cognitive dysfunction, indicating brain structure damage due to neuronal injury. Localized gray matter changes may result from hypoxia-related inflammation. Future research should explore the effects of interventions on these findings. Understanding these anatomical abnormalities is crucial for improving clinical interventions, prognosis, and therapeutic strategies.

## Data Availability

The raw data supporting the conclusions of this article will be made available by the authors, without undue reservation.
